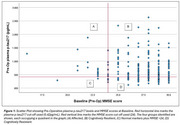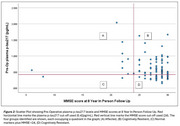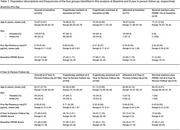# Using Plasma *p*‐tau217 to Define Cognitive Resilience in an Elective Hip and Knee Surgery Cohort

**DOI:** 10.1002/alz70856_105455

**Published:** 2026-01-07

**Authors:** Rebecca J Egerton, Aoife Sweeney, Bernadette McGuinness, Owen A. Ross, David Beverland, Anthony Peter Passmore, Amanda J Heslegrave, Henrik Zetterberg, Emily Bowman, Emma L Cunningham

**Affiliations:** ^1^ Centre for Public Health, Queen's University Belfast, Belfast, Northern Ireland, United Kingdom; ^2^ Mayo Clinic, Jacksonville, FL, USA; ^3^ Belfast Trust, Belfast, Northern Ireland, United Kingdom; ^4^ UK Dementia Research Institute at UCL, London, United Kingdom; ^5^ Department of Neurodegenerative Disease, National Hospital for Neurology and Neurosurgery, UCL Institute of Neurology, London, United Kingdom; ^6^ Hong Kong Center for Neurodegenerative Diseases, Hong Kong, Science Park, China; ^7^ Wisconsin Alzheimer's Disease Research Center, School of Medicine and Public Health, University of Wisconsin‐Madison, Madison, WI, USA; ^8^ Department of Psychiatry and Neurochemistry, Institute of Neuroscience and Physiology, the Sahlgrenska Academy at the University of Gothenburg, Mölndal, Gothenburg, Sweden; ^9^ Clinical Neurochemistry Laboratory, Sahlgrenska University Hospital, Mölndal, Västra Götaland län, Sweden; ^10^ Department of Psychiatry, University of Oxford, Oxford, United Kingdom

## Abstract

**Background:**

Plasma phosphorylated‐tau217 (pTau217) is a blood‐based biomarker of Alzheimer's Disease (AD) pathology. Cognitive resilience describes the presence of neurodegenerative pathology and preserved cognition. Cognitive resistance describes avoidance of neurodegeneration. This post hoc analysis of the Post‐Operative Delirium Belfast (PODB) cohort aimed to define cognitive resilience using plasma pTau217 and Mini Mental State Examination (MMSE) measures.

**Methods:**

PODB was an observational cohort study, investigating people aged 65 and over, without a diagnosis of dementia, undergoing elective hip and knee replacement between 2012‐2014 with subsequent long‐term follow‐up. In this analysis, pre‐operative plasma pTau217 was used as a marker of neuropathology. A cutoff of 0.42 pg/mL was used (Ashton NJ et al. 2024). Pre‐operative and 8‐year follow‐up MMSE scores were used as a marker of cognition. A score of ≥24 was considered preserved. Definitions used were: Cognitive resilience = pTau217 ≥0.42 pg/mL plus MMSE ≥24. Cognitive resistance = pTau217 <0.42pg/mL plus MMSE ≥24. Affected = pTau217 ≥0.42 pg/mL plus MMSE <24. Those with normal markers plus poor cognition were defined as pTau217 <0.42pg/mL plus MMSE <24. These four groups were identified at baseline and follow‐up, respectively. In this analysis, 231 participants were included at baseline, 104 at follow‐up.

**Results:**

Of the *n* = 231 participants included at baseline, *n* = 147/231 (64%) were cognitively resilient, *n* = 65/231 (28%) resistant, *n* = 17/231 (7%) affected, and *n* = 2/231 (<1%) had normal markers and MMSE <24. Of the *n* = 104 participants with follow‐up information included in this analysis, *n* =  61/104 (59%) were cognitively resilient, *n* = 33/104 (32%) were resistant, *n* = 8/104 (8%) affected, and *n* = 2/104 (<1%) had normal markers and MMSE <24. Cognitively resilient participants at baseline were younger (*p* = 0.011) and tended to be female (*p* = 0.977) compared to affected participants. At follow‐up, those who were cognitively resilient tended to be younger (*p* = 0.196) and female (*p* = 0.212) compared to affected participants (Table 1). Of those cognitively resilient at follow up, *n* = 59/61 (97%) of these participants were also cognitively resilient at baseline.

**Conclusions:**

Using pTAu217, at the cut‐off quoted, we found a high incidence of cognitive resilience in an older elective surgical cohort.